# Ethics of digital contact tracing and COVID-19: who is (not) free to go?

**DOI:** 10.1007/s10676-020-09544-0

**Published:** 2020-08-24

**Authors:** Michael Klenk, Hein Duijf

**Affiliations:** 1 Institute for Business Ethics, St Gallen, Switzerland & Delft University of Technology, Delft, Netherlands; 2 VU Amsterdam, Amsterdam, Netherlands

**Keywords:** COVID-19, Digital ethics, Fairness, Digital contact tracing, Active responsibility

## Abstract

Digital tracing technologies are heralded as an effective way of containing SARS-CoV-2 faster than it is spreading, thereby allowing the possibility of easing draconic measures of population-wide quarantine. But existing technological proposals risk addressing the wrong problem. The proper objective is not *solely* to maximise the ratio of people freed from quarantine but to also ensure that the composition of the freed group is fair. We identify several factors that pose a risk for fair group composition along with an analysis of general lessons for a philosophy of technology. Policymakers, epidemiologists, and developers can use these risk factors to benchmark proposal technologies, curb the pandemic, and keep public trust.

The SARS-CoV-2 pandemic has forced almost a third of the world’s population into some form of quarantine (Kaplan et al. 2020), causing severe rights-restrictions, as well as drastic economic, social, and psychological harms. Societies are seeking ways to return to normality. Evaluating ‘when’ to get back to normal is hard, but the question of ‘how’ is no less daunting (Walensky and Del Rio 2020). A significant proportion of SARS-CoV-2 infections (47%) occur before the onset of symptoms, which means that infected individuals will likely spread the virus unknowingly. These pre-symptomatic infections make traditional contact tracing approaches infeasible (Ferretti et al. 2020).

Digital tracing technologies have been proposed as a solution to manage pre-symptomatic infections by alerting individuals and others they have come into contact with in real-time of high-risk exposures, imposing a quarantine on the full contact chain. Singapore released such a contact-tracing app on March 20th 2020, and similar developments are currently underway in many other countries (Azevedo Silva 2020). Digital tracing elicits an individual’s self-reported health status and identifies individual’s contacts through smartphone-based proximity capture.

We argue in this article that, despite its intuitive appeal, digital tracing risks addressing the wrong problem, and in consequence, its employment raises pressing and hitherto unacknowledged concerns about fairness. There are significant risks that digital tracing apps will fail to sufficiently reduce the number of people in quarantine while introducing new psychological, social, economic, and political risks associated with such large-scale technological experiments. Our central claim is that digital tracing poses a fairness risk. In particular, because having a job that cannot be done from home is unequally distributed across social networks in society, existing societal inequalities may be perpetuated, and already vulnerable groups in society will bear disproportionately heavy costs because of digital tracing. This fairness risk is a symptom of asking ‘What technology?’ before asking ‘Why technology?’ to evaluate what problems need solving in the first place. Fairness is an important one, or so we argue. Hence, before societies implement digital tracing, they should consider these concerns and embed digital tracing in a suitable non-technical infrastructure (including, for example, sufficient testing capacities).

The article thus contributes to an improved problem definition and describes concrete factors that will have to be investigated to assess the efficiency and ethical legitimacy of digital tracing as a measure to contain the SARS-CoV-2 pandemic. Many of the factors that we mention are risks—empirical studies and structural models are needed to quantify their magnitudes.

We introduce the case for digital contact tracing in Sect. “The case for digital contact tracing”, present our criticism in Sect. “Against digital contact tracing”, and draw broader conclusions for digital ethics in Sect. “The perils of asking ‘what technology?’ before ‘why technology?’”.

## The case for digital contact tracing

This section introduces key epidemiological terms, makes precise the problems that need solving, and clarifies the kind of digital tracing in question. Apart from introducing the normatively salient points of the debate and providing valuable entry points for subsequent ethical discussions related to SARS-CoV-2, we demonstrate and motivate the relevance of fairness in adopting digital tracing as a response to SARS-CoV-2.

### What problems need solving?

In most countries, when SARS-CoV-2 was acknowledged, the opportunity for containment measures, like isolation of cases and their contacts, had passed. Community spread had occurred, and transmission chains could not be traced to isolate infected individuals and quarantine contacts. With containment forgone, governments switched to mitigation strategies to prevent overload of the healthcare system (Parodi and Liu 2020). Mitigation has been crucial to saving lives. While “a blunt and inconvenient tool,” it has proven to reduce and delay peak outbreaks and mortality rates (Walensky and Del Rio 2020, p. 1).

But mitigation is dreadful: it causes severe economic, social, and psychological harms (Calvo et al. 2020), and is prone to political abuse. The harmful effects of mitigation are exacerbated by economic factors: Poorer countries and those with weak healthcare systems have only limited means to remedy the harms of mitigation, e.g. by offering financial compensation to those harmed by the quarantine. In such instances, the harm to livelihoods is more significant and, thus, (more readily) outweighs considerations for saving lives (The Economist 2020b).

Therefore, societies must find ways to decrease safely the number of people in quarantine speedily, restore infringed rights, and avoid unnecessary economic, social, and psychological harms. Assuming that the success of mitigation measures eventually allows returning to a containment approach, the first two problems are as follows:

1. **‘Saving Lives’ Problem**: How can the spread of the virus be contained, at least to the extent that overloading ICUs is avoided?
2. **‘Saving Livelihoods’ Problem**: How can the ratio of free, unquarantined people in a society be maximised?^1^

However, these are not the only problems that need to be addressed. There is a further, hitherto unacknowledged problem that puts constraints and restrictions on possible solutions.^2^ Societies need to ensure that the costs of fighting the pandemic are allocated fairly, for both pragmatic and ethical reasons. Pragmatically, unequal allocations of costs, particularly to already disadvantaged groups, may further drive anti-democratic tendencies like nationalism and radicalism (cf. Fetzer 2019). Ethically, measures should benefit all parts of society equally, where inequalities ought to be justified so that they are to the greatest benefit of the least advantaged members of society (Rawls 1971). Hence, in resolving the lives vs livelihoods dilemma, a third problem transpires:

3. **‘Ensure Fairness’ Problem**: How can we ensure that the costs of the pandemic and its countermeasures are fairly allocated in society?

Ensuring fairness is of particular relevance in a discussion of digital tracing because several other technological solutions, such as AI-based technologies, have been shown to potentially exacerbate existing inequalities (e.g. Fuster et al. 2020).

A hierarchy of problems from saving lives, to saving livelihoods, to the fairness problem, suggests itself, with two relevant qualifications. First, there will likely be “excruciating” trade-offs between saving lives and saving livelihoods (The Economist 2020b), and questions about the constraints of maximising saving livelihoods vis-à-vis saving lives as well as the appropriate baseline. We will not discuss ethical questions arising in this context, because we address digital tracing as a proposal for a responsible innovation that aims to evade or at least minimise this trade-off (cf. van den Hoven 2013). Second, we take the fairness problem to put a pro tanto constraint on measures proposed to solve the first two problems (cf. Ross 1930). Although fairness considerations may thus justifiably be overridden, we should do everything we can to meet them. Hence, the forward-looking responsibility of governments is to solve the Saving-Lives and Saving Livelihoods problems *while* solving the Ensuring Fairness problem, too.

Next, we will explore the limitations of traditional contact tracing in solving the first two problems, and then turn to the promises of digital contact tracing.

### Limitations of traditional contact tracing

Contact tracing is a well-tested and often successful response as part of a containment strategy. It works by isolating infected individuals and quarantining their contacts and aims at stopping the spread of the virus by reducing the number of transmissions, both from symptomatic individuals and their contacts while minimising the impact on the larger population (cf. Ferretti et al. 2020, p. 4; Groch and Hope 2020). It has proven to be successful in several earlier occasions (cf. Klinkenberg et al. 2006; Landman 2020). For example, the 2003 SARS outbreak in China was controlled by contact tracing because the majority of transmissions occurred only after the onset of symptoms (Glasser et al. 2011).

What is now known about the epidemiological characteristics of SARS-CoV-2 makes traditional contact tracing infeasible. Let time-to-isolation be the timespan between an individual’s *onset of infectiousness* and the individual's isolation. People infected with SARS-CoV-2 are infectious up to 7 days before the onset of symptoms, and epidemiological research suggests that about 47% of transmissions originate from pre-symptomatic individuals (Ferretti et al. 2020). However, when the rate of transmission by pre-symptomatic individuals is high traditional contact tracing becomes increasingly infeasible (Peak et al. 2017; Fraser et al. 2004; Hellewell et al. 2020). Partly, this is because the *time-to-identification*, between the onset of infectiousness and becoming aware of it, is long. So, isolating every symptomatic individual will still be inefficient to contain the virus.

Another critical factor in the discussion about digital tracing and SARS-CoV-2 is the *time-to-quarantine-contacts* (TTQC) of infectious individuals, where ‘contacts’ are individuals within the transmission range of the virus. TTQC depends on the *notice of contact,* where contacts become aware of being near an infectious individual, and each contacts *time-to-quarantine* (e.g. return home, implement physical distancing). Notice of contact depends on the time the infectious person is informed of her infection and the *time-to-reach-all-contacts* of that person. The high ratio of pre-symptomatic infectiousness with SARS-CoV-2 will tend to prolong notice of contact a) because infectious persons are alarmed slowly and b) time-to-reach-all-contacts is prolonged because there will likely be more contacts that are harder and slower to trace. In a significant contribution, Ferretti et al. (2020) show that traditional contact tracing will not be able to contain SARS-CoV-2, assuming that traditional contact tracing has a TTQC between 1 and 3 days.

Success in solving the containment problem for SARS-CoV-2 thus depends on minimising the time-to-identification *and* the time-to-quarantine-all-contacts. The characteristics of SARS-CoV-2 means that the notification of infection will be late. But the time-to-reach-all-contacts can be shortened, and that is what digital tracing hopes to do.

### The promises of digital contact tracing

Digital tracing can—by design—improve contact tracing. Contacts are recorded, stored, and contacted digitally. Precious time to-reach-all contacts is saved, which, all else being equal, will shorten TTQC. The models of Ferretti et al. (2020) suggest that a shorter TTQC, plausible through digital contact tracing, makes containing the epidemic more likely. The models of Hinch et al. (2020) suggest that under plausible assumptions, digital contact tracing can significantly lower the number of people in quarantine to 30–50% of the population, significant gains relative to an approximate 100% mitigation regime that excludes only key workers.

Therefore, digital contact tracing promises to solve both the Saving-Lives and the Saving-Livelihoods problem by providing a means to substantially increase the number of free, non-quarantined individuals while avoiding an overload of the healthcare system. We stress that these inferences are not based on randomised controlled trials but epidemiological modelling informed by observational data. Decisions about digital contact tracing must be based on further corroborations of such studies.

Digital contact tracing can be implemented in several ways, to be distinguished by the *method of registering contacts* (e.g. GPS, Bluetooth, mobile phone data) and the *method of storing contacts* (e.g. de-centralised, on each user’s phone, or on a centralised server). We will focus on the variant discussed in the modelling studies, which is currently being evaluated by many governments, and thus most likely to be implemented: a smartphone app that registers contacts via Bluetooth and stores them locally on the user’s phone. When a user is alarmed of her infection, she triggers an alarm via the app that automatically alerts all of her registered contacts.

Use of such a digital tracing app probably has greater potential to resolve the problem of lockdown relative to digitally unaided, traditional contact tracing. We emphasise, however, that *greater potential* at solving the Saving-Lives- and the Saving-Livelihood problems *does not equal eventual success*, as we demonstrate in the next section.

## Against digital contact tracing

We will now argue that several risk factors need to be eliminated and several ethically significant open questions answered before governments can responsibly endorse digital tracing as a solution to the aforementioned three problems. A preponderance of behavioural and infrastructural risks associated with digital contact tracing casts doubt on its efficacy to Save Lives and to Save Livelihoods (in Sect. “Insufficient reduction of quarantined population while saving lives”). Moreover, there is significant risk that digital tracing fails to Ensure Fairness (in Sect. “Unfair composition of quarantined population”).

Before turning to our argument, it is essential to flag some assumptions and ethical issues that we will set aside in our analysis. Notably, we will pass over concerns about *privacy*. Promising proposals to mitigate privacy concerns in digital tracing are under development (Demirag and Ayday 2020; Bell et al. 2020; Raskar et al. 2020). We stress that opting for de-centralised, privacy-protecting approaches may further corroborate the problems we raise below, and that *some* privacy concern will likely remain. Besides, large-scale implementation of the app will create a risk of ‘mission creep’ (to wit, continued use of the app for other, perhaps nefarious goals), as well as abuse by ill-minded users (Anderson 2020). Finally, we will set aside technical questions pertaining to the reliability and ease of updating the app (particularly to adjust to new knowledge about SARS-CoV-2, e.g. about transmission routes), and how to ensure that contacts are reliably registered, without increasing false-positive rates too much (Newton 2020).

These factors represent significant risks *irrespective of whether the app resolves the Saving Lives, Saving Livelihoods, and Ensuring Fairness problems*. That these risks are significant and partly created by digital tracing (as opposed to being general risks associated with mitigation measures) is a crucial premise in our argument, and we will defend it throughout Sect. “Against digital contact tracing”. With pharmaceutical measures like a vaccine, elaborate testing would be required to establish the prevalence and degree of the vaccine’s ‘side-effects’ and the efficacy of the vaccine itself (cf. van de Poel 2020). No comparable testing regimes exist for running societal experiments like the introduction of digital tracing. If our argument about the risks associated with a digital tracing app hold, then these risks should be weighed even heavier in decisions about whether to implement digital tracing. With uncertainty about the efficacy of digital tracing and the risks associated with it, the downsides may well tip the balance *against* digital tracing.

We can now turn to our argument that there are several risks concerning the efficacy of digital tracing to Save Lives and Save Livelihoods while Ensuring Fairness.

### Insufficient reduction of quarantined population while saving lives

For digital contact tracing to meet the minimal aim of resolving the Saving Lives and Saving Livelihoods problems, several optimistic assumptions about *user behaviour* and the *available non-digital infrastructure* must hold. But there are considerable open questions. Below we outline these factors and urge policymakers, epidemiologists, and app developers to test for the assumptions.

First, a factor of major importance for the app’s efficacy is *user app-uptake*. About 80% of smart phone users in the UK, or 60% of the population, would have to use the app according to an unpublished model by Hinch et al. (2020). So, if more than 1 out of 5 smart phone users refrain from using the app, the efficacy is lost and a public health catastrophe will not be averted. Moreover, depending on locality, the access to suitable devices that can run the digital tracing app will differ, so that digital tracing may fail to be an efficient measure even if all smart phone users use the app. Uptake of digital tracing in Singapore, however, has been a meagre 16% (The Economist 2020a). All in all, this goes to show that a large uptake among all of society is *necessary* for the app to be an effective tool in combating the spread of the virus.^3^

But while app-uptake is crucial, and often discussed, another crucial user behaviour relates to *user discipline*, which will affect TTQC: How fast do symptomatic individuals report their infection and how fast do notified contacts go into quarantine?^4^ Although it is a clear advantage of digital tracing that it virtually decreases the *time-to-reach-all-contacts* to zero, there may be other hiccups in the process from a symptomatic person to quarantining their contacts. Ferretti et al. (2020, Fig. 3) show that every extra day it takes to isolate and quarantine contacts would drastically reduce the efficacy of contact tracing. Moreover, if the TTQC is 2 days or more, then their models reveal that it is uncertain whether contact tracing has any positive effect. This demonstrates that it is important to keep TTQC to a minimum and essential to keep it below 2 days.

But there are reasons to doubt that users will show the required discipline. For instance, there may be significant delays in reporting symptoms. It should not be assumed that users will immediately seek help and report their symptoms after the onset of symptoms, as a multitude of social and infrastructural factors have an impact (e.g. Pescosolido 1992). Moreover, the consequences of reporting symptoms for one’s contacts will likely affect user’s readiness to act—but it is unclear in which direction. Users may be quicker—being aware that their action helps to curb the virus—but could also slower, being unsure about their symptoms and thus hesitant to cause drastic consequences on their contacts (of which they will know at least some).

Individuals receiving a notification that one of their contacts is infected may also significantly delay going into quarantine. Empirical evidence would be desirable to investigate people’s readiness to comply with quarantine measures. A suggestion from social science is that people comply with current quarantine restrictions because of legal, social, and moral norms (cf. Bicchieri 2006; Bowles 2016). People’s compliance with legal and social norms, however, depends on those norms being enforceable, which is not a given with an app that alerts the user only privately (so that privacy considerations may hamper the effectiveness of digital tracing). It is an open empirical question whether relevant moral norms about using the app (understood as norms that *do not depend on being enforceable)* are sufficiently widespread in society to ensure efficacy of the app.

Second, the efficacy of the app will depend on *non-digital infrastructure*, notably the availability, reliability, and speed of tests. Test are required to reduce the number of quarantined people by testing individuals that have been ‘sent into quarantine’ by the app and to release them in case of a negative test result. The scenario with the largest number of reduction in the quarantined population discussed by Hinch et al. (2020) requires testing the index cases. They estimate that about 100.000 tests per day will be required in a population like the UK.

But, again, there are currently limitations for the non-digital infrastructure, and this yields reasons to doubt that the digital solution will resolve our issue. Tests must be available and deployable speedily to ‘app-quarantined’ patients to free uninfected people from quarantine and to reduce the toll on them. The expedience of such a system must be evaluated.

Proponents of digital tracing may invoke the precautionary principle. Greenhalgh et al. (2020)suggest that the precautionary principle may favour the use of face masks despite little evidence in their efficacy. One might defend the use of the app analogously. However, the analogy fails. Using face masks in futility creates rather limited costs while the gains might be high. In the case of tracing apps, the scale of risks is largely unknown.

Digital tracing offers an opportunity to minimise the hidden spread insofar as it promises to reduce the number of hidden contacts. Given the vital importance of speed and comprehensiveness in isolating and quarantining, such an opportunity should not be missed. Nevertheless, decision-makers should ensure that the other crucial factors in a digital tracing approach are in place, too.

Digital tracing apps can target a selected group of people for quarantine and thereby appeal to the hope that the draconian lock-down that we see in many countries today can be lifted. But how many people will be quarantined with digital tracing? For the sake of argument, let us consider the optimistic scenarios where digital tracing successfully averts a public health catastrophe, that is, it solves the *Saving Lives* problem.

Although there have not been many findings on this aspect of digital tracing, the simulations by Hinch et al. (2020) highlight the relevance of this issue. Their models show that under plausible assumptions, it is to be expected that roughly 30–50% of the population will be in quarantine when digital tracing is adopted.^5^ Of course, the more effective policies where symptomatic persons, their contacts, and all their families are quarantined yield the highest turn-in of 50%. So, even though digital tracing apps reduce the number of people in quarantine, it is an open ethical question whether the benefit of lifting the quarantine for a subset of our society outweighs the risks associated with the app (and with abandoning a lock-down).

### Unfair composition of quarantined population

We will now argue that digital tracing poses a risk to fairness and thus may fail to solve the *Ensuring Fairness* problem *even if* it solves the *Saving Lives and Saving Livelihood* problems. Even if digital tracing solves the saving lives and saving livelihood problems, it may disproportionately burden already disadvantaged groups with the costs of mitigating the crisis by, for example, quarantining them more frequently and yielding more personal costs associated with the digital tracing technology. Hence, the question is not just whether digital tracing solves the saving livelihoods problem but also how it can ensure that the costs of the pandemic and its countermeasures are fairly allocated in society.

It has already been observed that there is an unequal distribution with regards to the effects of the disease irrespective of the mitigation strategy. The costs of the SARS-CoV-2 pandemic are distributed unequally across countries and within societies. Within some societies, people of colour and migrants are disproportionately affected by the effects of COVID-19, for example by being represented disproportionately amongst COVID-19 deaths (Legido-Quigley et al. 2020); In the UK, health workers belonging to ethnic minorities die at higher rates (Barr et al. 2020). Similar inequalities emerge across countries: The World Bank finds that necessary mitigation measures will disproportionately affect poorer countries (Loayza 2020).

But digital tracing poses a hitherto unnoticed risk regarding fairness: Disadvantaged groups may carry disproportionately heavy costs of countermeasures. In particular, digital tracing may lead to unfair distributions of the costs of mitigation measures (already disadvantaged groups bear them disproportionately, e.g. by ending up in quarantine to a disproportionate degree).

Therefore, there is urgency in addressing the ‘Ensure Fairness ‘Problem when employing digital tracing technologies to alleviate the effects of the SARS-CoV-2 pandemic. Solving the ‘Saving Lives’ and ‘Saving Livelihoods’ Problems constitutes critical progress towards navigating the pandemic; however, policymakers ought to avoid that the resulting composition of the quarantined and freed groups in society differ in systematic ways that perpetuate existing societal inequalities. Solving this ‘Ensure Fairness’ Problem thus ensures that the costs and benefits of technologically determined quarantine are not be born disproportionally by (vulnerable) groups of the population without introducing possible compensation measures.

Mitigation measures will likely violate fairness considerations to some degree. To begin with, note that current models of mitigation measures assume that a significant part of the population—those at high risk—will remain quarantined. Realising this assumption into public policy may be justifiable, insofar as individuals at high risk from COVID-19 benefit the most from their quarantine and, at least in some measures, loose little. In contrast, quarantine should be avoided for those not at high risk, if it is possible to safely do so. But there is a significant risk that the composition of the quarantined population will be unfair.

On what grounds may we suspect that mitigation measures can yield systematic differences in the freed and quarantined group compositions? Ample empirical evidence from the social and economic sciences suggests that the structures of social networks in societies will play a critical role in determining behavioural interactions with any technological solution. Further, recent research finds that the fraction of work that can be done from home varies substantially across industries, with those associated with low incomes and precarious work arrangements least likely to be done from home. Both observations will likely interact and give reason to believe that digital tracing technologies will have significant distributional implications.

First, it is well known that similar individuals are more likely to form social ties of every type (McPherson et al. 2001). Such “homophily” in relationships has been shown to reduce the speed with which information spreads in society (Golub and Jackson 2012); to worsen inequality when effects of individual differences are propagated by social networks (DiMaggio and Garip 2012); and to constitute barriers to incentive design and new technology adoption given the presence of social norms and sanctions and, respectively, that word-of-mouth communication influences individuals’ opinions and beliefs (Jackson et al. 2015).

Second, the proportion of jobs that can be done from home varies significantly by industry (Dingel and Neiman 2020). Further evidence from the US suggests that relative to workers in high work-from-home (WFH) jobs, workers that cannot work from home are less likely to be white, college-educated, in receipt of employer-provided healthcare, more likely to be in the bottom half of the income distribution, and less likely to own their home (Mongey and Weinberg 2020). Importantly, these workers “are also less likely to have had stable jobs: more likely to have been unemployed in the last year, less likely to be employed full-time, and less likely to be employed in large firms” (Mongey and Weinberg 2020, p. 1).

The findings on homophily and WFH arrangements interact. Homophily suggests that disadvantaged workers are more likely to form social ties with others in similarly precarious arrangements. As these disadvantaged workers are less likely to be able to work from home, engaging in work means that they are at higher risks of becoming infected. Due to homophily, a digital tracing technology will have likely recorded more contacts of the disadvantaged worker with others that are disadvantaged and thus, in the case of selective quarantine, send a large proportion of the “disadvantaged worker network” into quarantine. It follows that, in addition to being disadvantaged already, these workers are more likely to bear the social, economic, and psychological ill effects of being quarantined.

Of course, in absolute terms, the workers’ situation would not be worse compared to the current situation in which the entire population is in quarantine. But their relative situation will be worsened because the well-off in a society will benefit a lot from the app. Someone working from the home office in suburbia will benefit from the app, because, for example, she will be able to frequent restaurants again. Compared to a waiter in downtown London who has to use the tube to get to work, she will be exposed to fewer people and less at risk of ending up in quarantine again. This particular negative consequence is not per se a potential design flaw in digital tracing, but it concerns any mitigation measure aimed at ‘freeing’ people from quarantine in justifiable ways.

In part, the fairness risks we identified are thus ultimately due to existing inequalities in a society and digital tracing as a specific mitigation measure does not worsen the situation compared to *other* mitigation measures that affect different groups in different ways. Even though some fairness risks are ultimately technology-neutral (and associated with discriminate as opposed to blanket mitigation measures), however, they *are* risks associated with digital tracing nonetheless and decision makers must take them into account in an evaluation of digital tracing as a solution to our three problems.^6^

Moreover, digital tracing itself contributes to fairness risks atop the general fairness risk associated with discriminate mitigation measures. Above we already identified several known risks with digital tracing, such as privacy risks and technological risks pertaining to false positive rates. Digital tracing risks burdening those with these specific technological risks unequally. For example, a high false positive rate may jeopardise fairness because quarantine is more costly for already disadvantaged groups and they may be more likely, under digital tracing, to end up in quarantine because they encounter more people. Digital tracing makes these negative fairness effects more likely; Compared to standard tracing measures, we can say that the technology itself *affords* unfair outcomes. And insofar as digital tracing affords these detrimental effects on fairness, it contributes negatively to the value of fairness (cf. Klenk 2020).

Therefore, there is a risk that digital tracing fails to solve the *Ensuring Fairness* problem, both because of digital tracing inherits fairness problems from non-digital discriminate mitigation measures and because it adds its own fairness risks. In times of crisis like the COVID-19 pandemic, governments substantially curtail their citizen’s freedom. They should use their power to rectify existing inequalities. Digital tracing will not be a genuine solution to our problem if it ignores or even perpetuates these problems.

## The perils of asking ‘what technology?’ before ‘why technology?’

Considerations about the rate of pre-symptomatic spread of SARS-CoV-2, and correspondingly longer *time-to-identify* infected individuals and *time-to-quarantine-all-contacts*, mean that traditional contact tracing fails and that digital contact tracing at least has the *potential* to be a solution. However, we have suggested that significant risks persist that may prevent digital contact tracing from solving either the Saving Lives-, Saving-Livelihoods-, and Ensuring Fairness Problems. On its own, digital contact tracing falls short.

The fact that digital tracing solutions are currently hastened to implementation without proper evaluation of the problems they ought to solve belies a problem eerily familiar in the history (and philosophy) of technology: overconfidence in technological solutions. Weinberg (1966), for example, suggested that technology can replace social engineering when it comes to the resolution of tragedy of commons situations as technology could simply *increase* the availability of the desired resource. However, Weinberg did not account for social factors that would influence the viability of his solution: he assumed that increased supply would solve issues, and ignored that demand would rise, too. The lesson from Weinberg, and for the case of digital tracing apps, is that a technological solution must not be seen independent from non-technical aspects.

Moreover, the case of digital tracing illustrates, and the speed with which governments around the globe have or will implement digital tracing suggests, that we, collectively, have overlooked the question of ‘why technology?’ and instead pursued answers to ‘what technology?’ (Klenk and Sand 2020). Before asking *what* technology we need (e.g. a centralised vs de-centralised app), we must ask *why* we need technology in the first place. Answering the latter question depends on a firm understanding of the problem that requires solving—which includes, as we argued, considerations about fairness. When it transpires that these ends can *also* be met by non-technological means, or that the available technological means are insufficient, then we will have to answer ‘why technology?’ in the negative (Floridi 2020).

Defending the urgent implementation of digital contact tracing suggests a confusion of duty and forward-looking responsibility. While the former requires action, the latter does not (van de Poel 2011). Governments have a duty of care toward their citizens. In the current situation, this duty necessitates the evaluation of ways to resolve the dilemma. However, they do not have an active, forward-looking responsibility to implement the app just yet.

## Conclusion

The SARS-CoV-2 pandemic confronts societies around the globe with three problems: Saving Lives while Saving Livelihoods, while also, as we argued, Ensuring Fairness. The costs of remedying the pandemic must, ethically and pragmatically, be distributed fairly in a society. Digital tracing has the potential to transcend the trade-off between saving lives and livelihoods by freeing people from quarantine while containing the virus. However, we noted significant risks to attaining these goals and also urged that digital tracing must ensure fairness. The considerations outlined above suggest that the feasibility of digital contract tracing in solving these problems, however, is far from clear. We, therefore, urge policymakers, epidemiologist, and developers to carefully consider the behavioural and infrastructural risk factors we outline above, which will determine whether digital tracing can help us fight SARS-CoV-2 responsibly.

## Footnotes

1 We understand the aim of maximisation against the baseline of solving the *Saving Lives* problem, i.e. we need to as many people in a society as possible *while* avoiding overloading ICUs. There is thus a hierarchy amongst problems, as we discuss further below.

2 Two manuscripts on the ethics of digital tracing and COVID-19 mention fairness only peripherally. Parker et al. (2020) emphasise fairness as a general factor in responding technologically to pandemics but we argue that digital tracing itself must contribute to fairness to be acceptable as a solution. Morley et al. (2020) mention accessibility to the app, which is a fairness-relevant factor, but leave out broader repercussions like access to the app that we discuss.

3 The efficacy of a digital tracing app partly depends on its uptake in a society, which raises a question about whether using the app should be mandatory or voluntary. Ultimately, this question also pertains to the efficacy of digital tracing as a solution to the three problems posed in Sect. “What problems need solving?” above. For reasons of space, we can only offer some preliminary thoughts on this particular question. Making it mandatory *may* alleviate worries about insufficient uptake to some extent but it will raise further ethical issues about personal autonomy and it poses the risk of citizens becoming increasingly resistant to governmental interventions if digital tracing is both mandatory and unsuccessful. Alternatively, incentivising the use of the app, perhaps financially or through a gamification approach, may offer a way to avoid the negative effects of mandatoriness. It is an open question, however, whether financial incentives may crowd out moral motivations for using the app, which would be a reason against using financial incentives, cf. Bowles (2016).

4 There are different approaches to initial exposure notification. An important distinction is between exposure notification based on a medically verified diagnosis of infection (we can call this the ‘authority report model’) and self-report of symptoms (the ‘self-report model). Hinch et al. (2020) focus on self-report models, and our discussion is primarily focused on this approach. Though the ethical issues differ to some extent, the concerns we raise ultimately apply to both approaches, insofar as testing for the virus will be initiated by users (i.e. a user has to seek medical attention to get a test).

5 Their study covers six contact-tracing policies and all except one are based on quarantining contacts after index cases have self-reported symptoms. It is to be expected that these numbers are lower for policies based on quarantining contacts after index cases have been tested positively.

6 *Discriminate* mitigation measures (i.e. those that target only parts of the population) raise general concerns about fairness because they affect individuals that are mostly not blameworthy for being ‘in scope’ for those measures *and* they perpetuate inequalities in the ways outlined above. So, from a perspective focused purely on fairness, quarantining the entire population may indeed be best. However, fairness considerations can be overridden and considerations about saving lives and saving livelihoods likely do override them to some extent.
